# The Role of Autopsy in Diagnosing Fatal Chest Injuries in Road Traffic Accidents: A Literature Review

**DOI:** 10.3390/diagnostics15060778

**Published:** 2025-03-19

**Authors:** Matteo Antonio Sacco, Maria Cristina Verrina, Saverio Gualtieri, Agostinho Santos, Bárbara Ferreira Mendes, Alessandro Pasquale Tarallo, Aurora Princi, Stefano Lombardo, Pietrantonio Ricci, Isabella Aquila

**Affiliations:** 1 Institute of Legal Medicine, Department of Medical and Surgical Sciences, University “Magna Graecia” of Catanzaro, 88100 Catanzaro, Italy; matteoantoniosacco@gmail.com (M.A.S.); mariacristina.verrina@studenti.unicz.it (M.C.V.); saverio.gualtieri@studenti.unicz.it (S.G.); alessandropasquale.tarallo@studenti.unicz.it (A.P.T.); aurora.princi@studenti.unicz.it (A.P.); stefano.lombardo@studenti.unicz.it (S.L.); ricci@unicz.it (P.R.); 2 Instituto Nacional de Medicina Legal e Ciencias Forenses, Jardim Carrilho Videira, 4050-167 Porto, Portugal; 3 School of Medicine and Biomedical Sciences, University of Porto, 4099-002 Porto, Portugal

**Keywords:** road accident, autopsy, chest trauma

## Abstract

Road accidents are one of the leading causes of death worldwide, with significant repercussions on public health and the global economy. Fatal accidents can cause injuries in various anatomical areas with different dynamics. The thorax is one of the main sites involved in fatal accidents, due to the presence of vital organs such as the heart and lungs. Protective devices, such as seatbelts and airbags, also play a fundamental role in preventing chest injuries. However, external examination is often insufficient to determine the extent of internal trauma, resulting in significant difficulties in reconstructing the accident dynamics. In particular, in the absence of an autopsy, it is difficult to determine whether the driver or passengers were wearing protective devices, such as seatbelts, at the time of the accident. Diagnosing injuries secondary to protective devices, such as airbags, can also be complex without this assessment. Through a review of the literature, this work analyzes the different types of thoracic trauma that can be found at autopsy, providing indications to the forensic pathologist for the examination of these injuries. This review highlights the importance of the autopsy examination as a gold-standard investigation in the analysis of thoracic trauma from road accidents, in order to evaluate with certainty the injuries that caused death, and to facilitate the reconstruction of the dynamics for judicial purposes. Finally, an analysis of postmortem radiological investigations and of the role of protective measures in these events, such as the seatbelt and airbag, is provided.

## 1. Road Traffic Accidents

Road traffic accidents (RTAs) remain one of the leading causes of death worldwide, with thoracic trauma being a significant contributor to mortality in these events [1]. The number of fatalities and injuries resulting from road traffic accidents is staggering, with approximately 1.19 million people losing their lives each year due to these incidents [1]. According to the literature, in 2016 alone, 1.35 million people died in road traffic crashes, leaving millions more with serious injuries that often lead to long-term adverse health conditions [2]. The statistics reveal a disproportionate impact on low- and middle-income countries, which accounted for 93% of road traffic injury-related mortality in 2019, equating to an estimated 1.3 million deaths [2]. It is evident that the global burden of road traffic accidents is severe, affecting drivers, passengers, pedestrians, and cyclists alike [3].

From a psychological point of view, it is also important to consider the impact of the accidents on the lives of the survivors and on the families of the deceased. Psychological problems such as depression, phobias, anxiety, and post-traumatic stress disorder (PTSD) are common among road traffic accident survivors [4]. Studies have shown that experiencing or witnessing a severe auto accident can lead to PTSD, crippling phobias, uncontrollable anxiety, and profound sadness [5]. The death of another individual in the accident is a significant predictive factor for PTSD, especially if the survivor has a history of depression or prior traumatic experiences [6]. These psychological consequences can severely affect an individual’s quality of life.

The economic costs associated with road traffic accidents are equally alarming, placing a significant financial strain on both individuals and nations. On a global scale, road traffic crashes cost countries about 3% of their gross domestic product (GDP) [7]. This financial burden is even more pronounced in low- and middle-income countries, where the costs can exceed this average. In economic terms, the injury-related costs amount to about 1% of the gross national product (GNP) in low-income countries and 1.5% in middle-income countries [8]. The European Union (EU) estimates the socio-economic cost of fatal, serious, and minor injuries, including intangible elements, to be about 2% of its countries’ GNP [9]. For instance, alcohol-involved crashes in 2019 resulted in 14,219 fatalities, 497,000 injuries, and USD 68.9 billion in economic costs, accounting for 20% of all road traffic crash-related expenses [10].

Many fatal accidents are associated with major trauma involving the thorax, with significant injuries. In this work, we explored, from a forensic point of view, the types of thoracic trauma, the most frequent injuries to the organs involved, and the possible dynamics in these traumas. To this end, in this study, we propose a literature review focused on thoracic traumas, analyzing the role of the forensic pathologist and the prevention measures that can be implemented. Unlike the previous literature, which has often focused on either autopsy or radiological imaging in isolation, this work highlights the complementary role of postmortem computed tomography (PMCT) in enhancing forensic diagnostics. This study reinforces the necessity of a multimodal forensic approach. Additionally, this work provides an in-depth medico-legal perspective by analyzing how injury patterns correlate with crash dynamics and occupant positioning, an area often overlooked in the forensic literature. The findings emphasize the importance of rib fracture distribution in determining seatbelt usage, airbag deployment effects, and potential secondary injuries from safety devices. This forensic insight is crucial for accident reconstruction and for differentiating primary impact injuries from post-impact trauma.

Another innovative element of this study is its emphasis on pedestrian fatalities. While many forensic studies focus on vehicle occupants, this review underscores that pedestrians suffer unique thoracic injury patterns, particularly involving posterior rib fractures and direct cardiac contusions from high-velocity impacts. This perspective is valuable for refining forensic protocols in evaluating pedestrian fatalities and guiding urban traffic safety measures aimed at reducing fatal chest injuries. Finally, this work advocates for the standardization of forensic protocols in thoracic trauma assessment by proposing an integrated three-stage autopsy approach, which includes external examination, systematic PMCT scanning, and detailed internal dissection with histological analysis. This proposed protocol enhances forensic accuracy and sets a new benchmark for evaluating thoracic trauma in road traffic fatalities.

## 2. Materials and Methods

This study was conducted as a narrative review aimed at analyzing thoracic injuries in RTAs, with a focus on forensic autopsy findings. A literature search was performed using PubMed NCBI, applying the following keywords: (“road traffic” OR “pedestrian”) AND (“Thoracic” OR “chest”) AND (“autopsy” OR “postmortem”). These keywords were selected based on the forensic relevance of thoracic trauma in traffic-related fatalities, and they were used to identify peer-reviewed studies, systematic reviews, and case reports published in the last two decades.

The inclusion criteria were as follows:

Studies analyzing autopsy findings in thoracic trauma from RTAs; research focusing on the correlation between injury distribution and occupant position (driver, passenger, pedestrian); studies investigating the use of postmortem imaging (PMCT) in forensic assessment of thoracic trauma; publications discussing injury mechanisms, accident reconstruction, and forensic protocols.

The exclusion criteria included studies focusing solely on survivors of thoracic trauma without forensic autopsy correlation, experimental animal models, and studies lacking a detailed methodology. This review aimed to highlight current forensic challenges in diagnosing thoracic trauma, the role of postmortem imaging, and the need for standardized forensic protocols.

## 3. Epidemiology of Chest Trauma

Studies have shown that thoracic trauma is one of the most commonly reported fatal injuries in RTAs, with rib fractures occurring in up to 81% of cases, particularly among elderly victims [11]. Notably, the pattern and severity of these injuries vary based on factors such as vehicle type, occupant age, and use of safety devices. Autopsy-based studies indicate that, in fatal RTAs, rib fractures, lung lacerations, and aortic ruptures are among the most common findings [12]. The incidence rates of chest injuries in road traffic accidents vary significantly across different regions and countries. In a global context, traumatic chest injury accounts for approximately 10–15% of all trauma-related hospital admissions, highlighting its substantial burden on healthcare systems worldwide [13]. This high prevalence rate is reflective of the overall frequency of road traffic crashes, which are a leading cause of such injuries. For instance, in some countries, road traffic crashes account for 80.4% of chest injuries, with other causes, like assaults and falls from height, contributing far less [14]. A recent autopsy-based study in Addis Ababa, Ethiopia demonstrated that 51% of road traffic accident victims had thoracic injuries, highlighting the prevalence and forensic significance of these traumas [15]. Similarly, a study in India found that combined thoraco-abdominal injuries occurred in 83.75% of victims, emphasizing the need for detailed forensic assessment. [16]. Among elderly victims, rib fractures were present in 76% of cases, confirming that older individuals are particularly vulnerable to severe thoracic trauma.

When comparing chest injury rates between urban and rural areas, a notable disparity emerges. Blunt chest trauma constitutes 47.8% of all injuries in urban regions, whereas in rural areas this figure escalates to 83.3% [13]. This stark contrast can be attributed to differences in road conditions, traffic volumes, and healthcare accessibility between urban and rural settings. Moreover, the frequency of road traffic injuries appears to be homogeneous in both urban and rural hospitals [17]. Demographic factors such as age, gender, and pre-existing conditions significantly influence the prevalence of chest injuries in RTAs. For instance, the highest incidence of thoracic injuries in female patients is observed in the 70–79 years age group, while in males it is most common in the 20–29 years age group [18]. Gender differences also play a role, with men typically experiencing a higher rate of serious chest injuries due to higher exposure to high-speed collisions [19].

Most patients affected by these injuries are car occupants, representing 52.3% of the cases [18]. This demographic trend indicates that individuals traveling in cars are particularly at risk, possibly due to factors such as seatbelt usage, vehicle speed, and crash dynamics. Demographic factors could play a crucial role in influencing chest injury rates from road traffic accidents. The literature therefore emphasizes the importance of analyzing cases considering some standard parameters, such as age, sex, and the geographical area where the accident occurred, in order to evaluate the phenomenon in a homogeneous way and identify suitable safety measures. Understanding these factors is essential for developing tailored interventions aimed at reducing the incidence of chest injuries in specific population groups.

## 4. Medico-Legal Analysis of Chest Trauma

When comparing chest injuries to other types of injuries sustained in road traffic accidents, thoracic trauma stands out as a major cause of mortality among trauma patients [20]. Chest wall injuries emerge as the most frequent thoracic injuries, accounting for 62.1% of cases, with multiple rib fractures constituting 52.4% of these injuries [21]. Furthermore, road traffic crashes rank as the leading injury mechanism, responsible for 41.4% of all chest injuries [20].

Rib fractures are the most common type of injury associated with trauma to the thorax, frequently resulting from road traffic accidents [20]. Recent autopsy studies demonstrate that rib fractures not only correlate with the severity of impact but also provide critical forensic clues regarding seatbelt use, airbag deployment, and crash dynamics. In elderly pedestrians, rib fractures are even more prevalent (up to 81%), likely due to decreased bone density. Moreover, autopsy findings indicate that rib fractures frequently coexist with pulmonary contusions and hemothorax, emphasizing the need for a comprehensive forensic approach that includes imaging techniques such as PMCT to assess fracture extent and associated injuries [15]. This type of injury can be particularly severe when multiple ribs are broken, leading to flail chest [22]. Flail chest is a traumatic condition in which three or more ribs are fractured in at least two locations, resulting in a segment of the chest wall that moves paradoxically during respiration [22]. This paradoxical movement can severely impair respiratory function and complicate the clinical management of the patient. Chien et al. retrospectively examined a total of 174 patients who were admitted to a hospital in Taiwan with thoracic trauma and multiple rib fractures [23]. The authors reported that the main traumatic mechanism was traffic accidents (58.6%), especially those involving motorbikes. The authors reported that the number of displaced rib fractures had a high specificity for predicting pulmonary complications, while patients with fewer than three fractures and without displacement had a better prognosis [23]. In this regard, various scores have been proposed in the literature, including the RibScore [24]. The forensic analysis during the autopsy therefore requires particular attention to rib fractures, with evaluation of the fracture site (anterior, lateral, and posterior arch), the number, and the site (left, right, bilateral). This evaluation is essential in order to understand the extent of the impact on the thorax, the presence of any safety measures at the time of the accident (such as the seatbelt and airbag), and the dynamics of the trauma. We suggest that three evaluations be carried out during the autopsy for rib fractures: the first with the thorax still closed during the external examination, in order to evaluate the possibility of traumatic crushing of the chest; the second after opening the sternum, in order to examine the fractures’ topography with respect to the position of the organs still in situ and evaluate any laceration action of the ribs on the organs; finally, it is essential to perform the third after emptying the thoraco-abdominal organs, in order to better examine the extent of the fractures and visualize trauma to the posterior arches.

**Figure 1:**
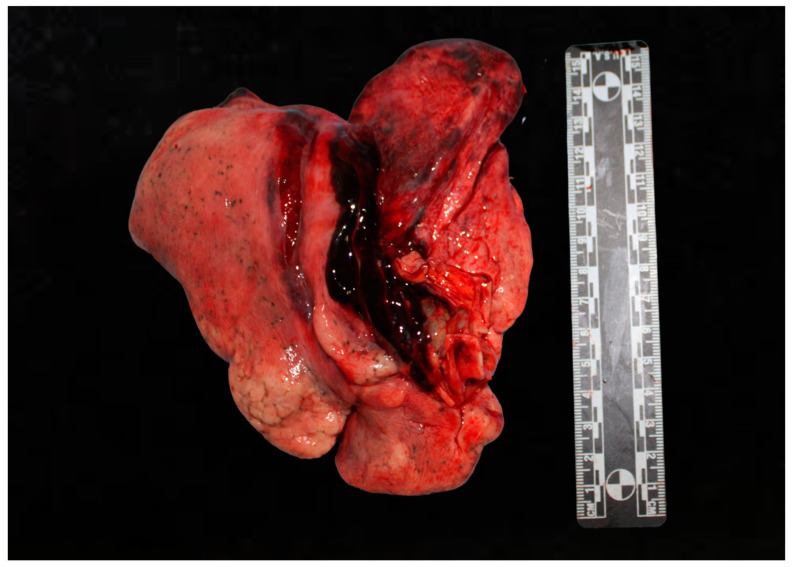
Pulmonary hemorrhage.

Pulmonary contusions and lacerations are other significant injuries that can occur following blunt chest trauma, often as a result of high-energy collisions in road traffic accidents [25]. A pulmonary contusion is characterized by injury to the lung parenchyma without any lung or vascular lacerations, leading to edema and hemorrhage within the lung tissue [26]. This type of injury is frequently observed following blunt chest trauma and can significantly impact lung function, leading to complications such as hypoxia and respiratory distress [27]. On the other hand, pulmonary lacerations involve tears in the lung tissue that can fill with blood, forming lung hematomas that are visible on radiographic imaging [28]. Both pulmonary contusions and lacerations require prompt medical evaluation and management to mitigate the risk of further complications and to stabilize the patient’s respiratory status. Reddy et al., in an autopsy-based study, analyzed a sample of 100 subjects who died following a road accident in the period between November 2008 and May 2010 [29]. The authors demonstrated that the lungs are the organs most involved in thoracic trauma (92.3%), followed by the heart (7.6%) (Figure 1).

The analysis of the forensic pathologist in these cases must therefore be concentrated on the analysis of the pleural cavities after the opening of the chest. In particular, it is necessary to examine the presence of any effusion in the chest, describe its characteristics macroscopically (hematic, serous, sero-hematic), and quantify its volume. Any lung lesions should be highlighted first in situ and then after sectioning the organ. Particular attention should also be paid to the analysis of the pulmonary hilum, evaluating the extent of the traction mechanisms undergone by the organ. It is also important to evaluate lesions such as pneumothorax or pulmonary atelectasis. Often, in the case of lacerations, the lung appears collapsed, and it is possible to appreciate areas of pulmonary hemorrhage in the parenchyma.

Cardiac injuries and traumatic aortic ruptures represent some of the most life-threatening outcomes of blunt chest trauma from road traffic accidents. Traumatic aortic rupture, for instance, is the second most common cause of death in victims of blunt chest trauma, accounting for a significant proportion of fatalities [30]. This injury typically occurs at the level of the aortic isthmus and is often fatal if not treated immediately [31]. Ye et al. analyzed a group of 27 road accidents involving the thoracic aorta, with particular reference to sex, age, vehicle used, and type of injury [32]. The authors highlighted that aortic injuries are more frequent in male subjects aged between 31 and 70 years, and that the most frequently associated vehicle is the motorcycle [32]. Such traumas are often accompanied by lung injuries and rib fractures. Generally the site of trauma is the ascending aorta, followed by the aortic arch and thoracic aorta. The most frequently identified injury was rupture of the thoracic aorta (63%), followed by intima and media injuries. The authors highlighted how the proximal part of the ascending aorta is the anatomical area most at risk of rupture. This is determined by the greater mobility of this anatomical site (isthmus) compared to the thoracic and abdominal aorta. Chellasamy et al. have highlighted how traumatic lesions of the aorta represent 18% of deaths related to road accidents, with a high mortality rate, being the second-leading cause of death from accidents [33]. In fact, only 15% of patients reach the hospital, of whom approximately 30% die within 6 h and 20% within 24 h if the diagnosis is delayed.

**Figure 2:**
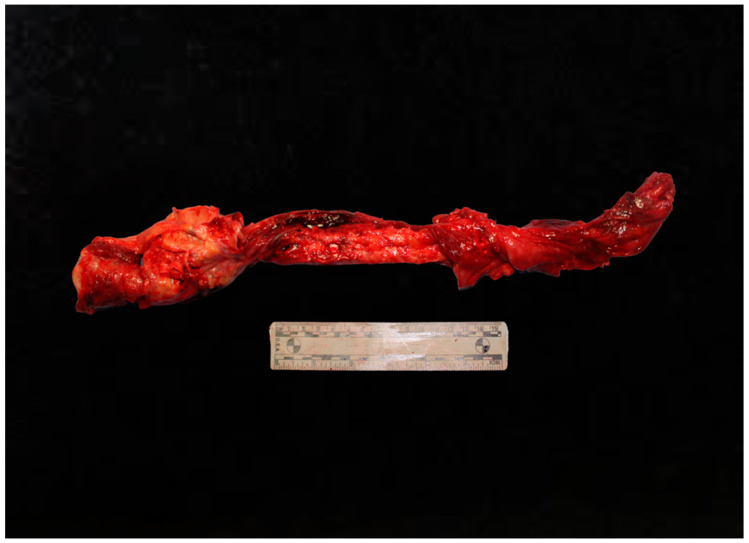
Aortic trauma.

In these cases, it is essential that the medical examiner performs an accurate analysis of the aorta at the opening of the pericardium. Generally, the aorta injury at its origin on the isthmus is accompanied by the presence of hemopericardium with clear signs of hemorrhagic infiltration at the autopsy. In particular, the presence of hemopericardium must be documented, its volume must be quantified, and it is necessary to analyze whether it is fluid or coagulated blood. When the hemopericardium is cleaned, it will therefore be necessary to proceed with the evaluation of the rupture point. It is advisable to always perform the analysis of such traumas in situ before the section of the heart, in order to avoid altering the identification of the hemorrhagic source. Furthermore, where there is lamination with the creation of false lumens, the pathologist should highlight this alteration in situ. It is also important to analyze the degree of atherosclerosis of the aorta and the presence of any previous comorbidities that could make rupture easier, such as aortic aneurysm (Figure 2).

**Figure 3:**
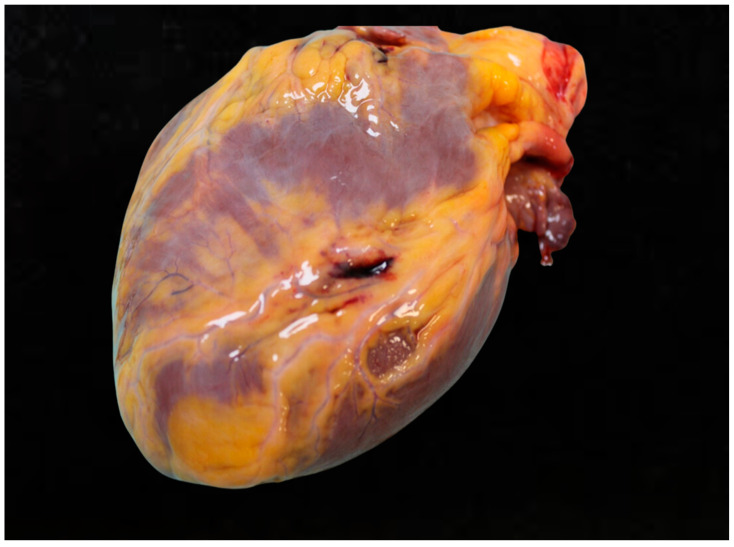
Cardiac injury.

Blunt cardiac injury (BCI) refers to the damage inflicted on the heart due to blunt trauma, often resulting from road traffic accidents. Myocardial contusion refers to the injury sustained by the myocardium following blunt chest trauma. Traffic accidents frequently cause cardiac contusions due to a direct blow to the chest [34]. The forceful impact of a collision can result in various forms of cardiac injury, including transmural rupture of a cardiac chamber and myocardial rupture [35]. These injuries are particularly common in high-speed motor vehicle accidents, where the incidence of blunt chest trauma is continuously increasing [21]. The severity of the injuries can vary widely, depending on the nature and force of the impact. Deceleration forces are another critical mechanism leading to cardiac injuries during road traffic accidents. Blunt cardiac trauma (BCT) often occurs due to rapid deceleration, where the sudden halting of the vehicle causes internal organs like the heart to continue moving forward, resulting in injury. Deceleration trauma is frequently observed in collisions involving motor vehicles, all-terrain vehicles (ATVs), and scooters [36]. Such injuries can lead to serious complications, including cardiac contusions and other forms of blunt cardiac trauma, contributing significantly to the morbidity and mortality associated with road traffic accidents [37]. Compression by safety devices, while intended to protect passengers, can sometimes contribute to cardiac injuries in road traffic accidents. The use of seatbelts and airbags, for instance, can result in mechanical chest compression, which may lead to cardiac contusions or other forms of blunt cardiac trauma [38]. Although these safety devices are crucial in reducing overall fatalities, they can inadvertently cause trauma to the chest and heart during significant impacts. Heart valve injuries, particularly those involving the aortic valve, have been increasingly reported in the context of road traffic accidents [38]. These injuries can lead to severe cardiac dysfunction and necessitate rapid diagnosis and intervention to prevent mortality. The high incidence and severity of these injuries underscore the need for rapid transport, resuscitation, and specialized trauma care to improve survival rates and outcomes for affected patients (Figure 3).

From a forensic perspective, traumatic injuries to the heart may be accompanied by pericardial rupture. In these cases, the forensic pathologist documents laceration of the pericardium with exposure of the heart in the thorax. Often, these are major injuries, characterized by high levels of injury, in which the laceration of the pericardium is accompanied by traumatic injury to the large cardiac vessels or injury to the heart itself. In other cases, the pericardium is intact, but other injuries may still be seen, such as cardiac contusions or, in more severe cases, cardiac rupture. In the case of an intact pericardium with cardiac rupture, the most prevalent injury mechanism is cardiac tamponade. In these cases, the forensic pathologist must document (1) the extent of the pericardial lesion, (2) the number and size of cardiac contusions, and (3) the size of the cardiac rupture area. This analysis must always be accompanied by standard measurements of heart size, diameters, and weight.

Moreover, in road accidents, a correct analysis of the heart is also essential in order to exclude any comorbidities that may have played a role in the accident, such as acute myocardial infarction or other acute complications. For this reason, it is important that the pathologist analyzes the integrity of the coronary arteries and evaluates the presence of any ischemic lesions. Generally, in cases of sudden cardiac death not related to the accident, the traumatic injury is reduced on both external and internal examination, and it appears incompatible with death. Autopsy studies have shown that coronary artery injuries, while rare, are a potential complication of blunt chest trauma, with an incidence of around 2% in all cardiac trauma cases [39].

Finally, thoracic spine injuries are often a result of high-energy trauma, such as motor vehicle accidents, leading to fractures or dislocations [40]. In these cases, the forensic pathologist must locate the fracture site using postmortem radiological investigations. This evaluation must be completed by direct visualization of the fracture after evisceration of the thoracic organs. The size of the fracture must be measured, as well as the depth and degree of spinal cord injury.

## 5. Mechanisms of Injury in Road Traffic Accidents

Impact forces in road traffic accidents often result in blunt trauma, which can lead to severe internal injuries without obvious external signs. High-energy impacts can cause significant damage to the thoracic region, including conditions such as flail chest, pneumothorax, hemothorax, lung contusions, ruptured diaphragm, and lacerations of the great vessels [18]. These injuries are frequently life-threatening and contribute significantly to traumatic mortality among accident victims [1]. Blunt chest trauma is commonly observed in motor vehicle accidents. Despite the lack of visible external injuries, the internal damage can be extensive, necessitating prompt medical attention and, often, surgical intervention [22].

Penetrating injuries resulting from debris or vehicle components are another critical aspect of thoracic trauma in road traffic accidents. These injuries tend to occur when sharp objects, such as glass shards or metal fragments, penetrate the chest cavity, causing severe and often fatal damage to internal organs [4]. Penetrating chest trauma is associated with a higher mortality rate compared to blunt trauma, and its incidence varies significantly depending on geographic and urban settings [23]. The severity and complexity of these injuries demand immediate and advanced medical intervention to prevent complications such as infections, excessive bleeding, and damage to critical structures like the heart and major blood vessels [24].

**Figure 4:**
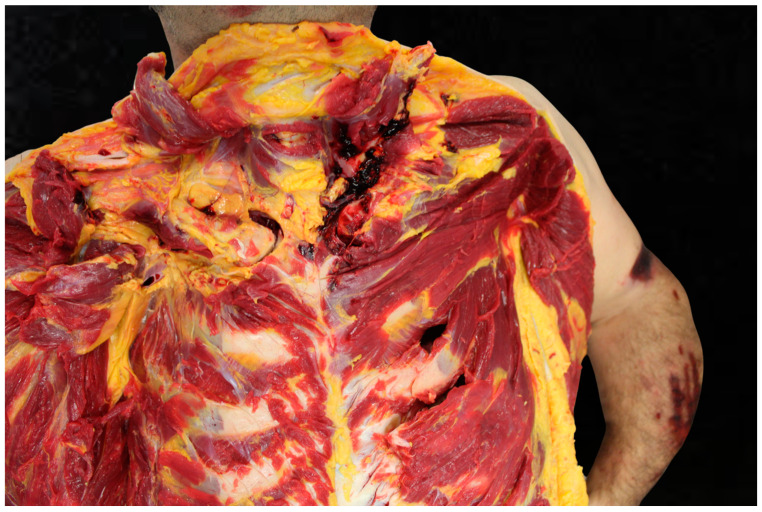
Neck injury.

**Figure 5:**
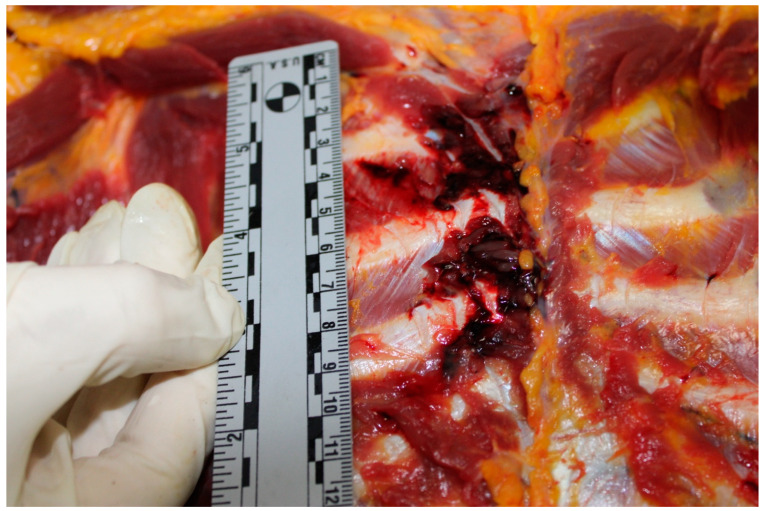
Sternal trauma.

Secondary injuries from seatbelts and airbags are also a significant concern in road traffic accidents. Seatbelt-related chest injuries often manifest as bruises and contusions, which occur due to the high force exerted during a collision. These contusions can span the chest, stomach, and neck, frequently forming a tell-tale seatbelt-shaped bruise that should prompt an immediate visit to the emergency room [1]. The deployment of an airbag can also cause bruising, as the sudden impact can compress the chest against the seatbelt, leading to contusions that might seem minor but could signify deeper tissue damage. Additionally, chest contusions are sometimes downplayed as mere bruising; however, they can indicate more severe underlying injuries, such as a myocardial contusion or bruised heart, which can range from a small bruise to a severe condition [3,4]. Rib fractures are another common type of chest injury resulting from seatbelt use during vehicular accidents. The force applied by the seatbelt during a crash can lead to multiple fractures in the ribs, especially in areas where the belt comes into direct contact with the body [5]. Research has shown that both seatbelt use and airbag deployment independently contribute to rib fractures, highlighting the significant impact of these safety devices under certain conditions [41]. Interestingly, simulations with adult models have predicted no rib fractures under a wide range of restraint settings, suggesting that the risk could be mitigated with optimal seatbelt design [42]. However, real-world incidents frequently demonstrate that the restraining loads transmitted via the seatbelt are a major cause of rib fractures [42]. Internal organ damage is a severe consequence of chest injuries from seatbelts, often occurring alongside visible bruising and rib fractures. The classic “seatbelt sign”, characterized by bruises or lacerations, can indicate internal injuries in about 30 percent of cases [43]. This sign is crucial, as it alerts healthcare providers to possible underlying damage to organs such as the lungs, heart, or even the liver and spleen. Thoracic injuries due to seatbelt force can include pulmonary contusions and, more rarely, myocardial contusions, adding further complexity to the medical assessment [43]. In some cases, the impact can lead to occult thoraco-abdominal trauma, which might not be immediately evident but could pose significant risks if not promptly diagnosed and treated [44] (Figure 4 and Figure 5).

Abrasions and burns are common chest injuries resulting from airbag deployment during a car accident. When an airbag deploys, it inflates rapidly, which can cause surface abrasions and burns due to the friction and heat generated by the airbag material [45]. These injuries can be both physical and chemical, with the latter caused by the chemicals used to deploy the airbag [46]. Although less common, burns from airbag deployment represent about 7–8% of airbag-related injuries [46]. It is important to note that while airbags are designed to reduce the overall risk of injury and death in motor vehicle accidents, they can still cause harm during their deployment [45]. Sternum fractures are another significant chest injury associated with airbag deployment. These fractures primarily result from blunt, anterior chest wall trauma and deceleration injuries, with an incidence rate of 3% to 6.8% in motor vehicle accidents [47]. The energy transferred to the chest during a high-speed collision can be substantial, leading to a sternal fracture. Even in moderate-speed accidents, a person’s chest or sternum can suffer severe damage from the impact with the airbag, steering wheel, or seatbelt. For example, there have been cases where a patient sustained a sternal fracture while being restrained by a seatbelt on the passenger side of a car during a moderate-speed collision [48]. Lung injuries are another potential consequence of airbag deployment. When an airbag inflates upon impact, the force exerted on the chest can lead to lung contusions or more severe conditions such as pneumothorax or hemothorax. Airbags, while reducing the overall risk of fatal thoracic injuries, can still cause significant trauma to the lungs, particularly in high-speed collisions. Chest injuries like contusions and bruising are commonly associated with airbag deployment, highlighting the dual nature of airbags as both protective devices and potential sources of injury. It is crucial to understand the balance between the benefits and risks of airbags in order to enhance vehicle safety measures [45].

## 6. Thoracic Injuries in Relation to Driver, Passenger, and Pedestrian Positioning

The distribution and severity of thoracic injuries in RTAs are significantly influenced by the position of the victim within the vehicle. Studies indicate that drivers and front-seat passengers experience different thoracic injury patterns compared to rear-seat occupants, primarily due to differences in restraint system interaction, steering wheel impact, and vehicle structure deformation upon collision [11].

Drivers are at higher risk of thoracic injuries caused by direct impact with the steering wheel, especially in cases where seatbelts are not worn or fail to function properly. Sternal fractures and anterior rib fractures are commonly observed in drivers due to the compression force between the thorax and the steering column, particularly in high-speed frontal collisions. A study on fatal motor vehicle accidents found that up to 62% of drivers with severe chest injuries sustained direct trauma to the sternum and anterior ribs, frequently leading to cardiac contusions and hemopericardium [49]. Additionally, in high-impact crashes, aortic ruptures at the isthmus are more prevalent in drivers, likely due to sudden deceleration forces affecting the upper body.

Front-seat passengers, while similarly exposed to frontal impact, exhibit slightly different injury patterns due to seatbelt and airbag deployment dynamics. Lateral rib fractures and lung contusions are more frequent in front-seat passengers due to sideward motion upon impact, especially in offset frontal crashes where the passenger is thrown against the door or center console. Furthermore, airbag-related injuries such as sternum fractures and pulmonary contusions are more commonly observed in front-seat passengers shorter than 160 cm, as improper airbag deployment timing increases the risk of direct thoracic trauma [49].

Rear-seat passengers, particularly those who do not use seatbelts, often sustain more severe thoracic trauma due to uncontrolled forward propulsion against the front seats or dashboard. Multiple rib fractures and severe lung injuries are more frequently observed in unrestrained rear passengers, with higher mortality rates linked to flail chest and hemothorax. Additionally, forensic studies have shown that rear passengers tend to suffer posterior rib fractures more frequently than front-seat occupants, likely due to hyperflexion of the thorax upon impact.

From a forensic perspective, the location and pattern of thoracic injuries can provide critical clues for accident reconstruction. The presence of classic seatbelt signs (bruising across the chest and abdomen), rib fracture distribution, and the presence of airbag-induced trauma can help determine whether the victim was wearing a seatbelt, sitting in the driver or passenger position, or was subject to secondary impact forces.

Pedestrians represent one of the most vulnerable groups in RTAs, often sustaining severe thoracic injuries due to direct high-energy impacts with vehicles. Unlike vehicle occupants, who benefit from safety mechanisms such as seatbelts and airbags, pedestrians are unprotected and experience direct trauma from both the initial impact with the vehicle and secondary impact with the road surface [12]. Autopsy studies indicate that rib fractures, pulmonary contusions, and aortic injuries are among the most frequently observed thoracic injuries in fatally injured pedestrians.

The pattern of thoracic trauma in pedestrians is highly dependent on the height and shape of the vehicle, the speed of impact, and the victim’s body position at the time of collision. Studies have shown that pedestrians struck by cars tend to sustain lateral and posterior rib fractures, as the vehicle’s hood or windshield exerts compressive forces on the chest upon impact [12]. In contrast, pedestrians hit by larger vehicles, such as trucks or buses, often experience more extensive thoracic trauma, including multiple bilateral rib fractures and severe lung lacerations, due to the greater force of impact and secondary crushing injuries.

Another critical factor influencing injury patterns in pedestrians is age. Forensic investigations have demonstrated that elderly pedestrians exhibit a higher prevalence of multiple rib fractures and flail chest, even in low-speed collisions, likely due to decreased bone density and increased fragility. Conversely, younger pedestrians are more prone to high-energy blunt thoracic trauma, often presenting with cardiac contusions, aortic rupture, and traumatic lung herniation.

## 7. The Role of Autopsy Investigations in Diagnostics of Chest Trauma from RTAs

Autopsy plays a crucial role in identifying internal injuries that may not be apparent through external examination alone. Studies comparing autopsy and PMCT indicate that PMCT is superior in detecting fractures and gas accumulations (such as pneumothorax), whereas autopsy remains more effective for assessing soft tissue and vascular injuries [50]. This suggests that a combined approach using both autopsy and imaging techniques may optimize forensic diagnostics in fatal RTAs. For instance, in cases of thoracic trauma from road traffic accidents, internal injuries such as rib fractures, lung contusions, or heart lacerations can be conclusively identified through a detailed autopsy [18]. This comprehensive examination enables medical professionals to document the full extent of injuries, which is essential for understanding the mechanisms of trauma. Furthermore, internal injuries are often correlated with specific types of external trauma, providing insights into the dynamics of the accident. Without an autopsy, these internal injuries might remain undetected, leading to incomplete or inaccurate conclusions regarding the cause of death. The correlation between external and internal injuries is another significant aspect that autopsies help elucidate. External injuries often provide initial clues about the nature and severity of the trauma experienced by the victim. However, it is through autopsy that a clearer picture emerges, linking these external signs to underlying internal damage [51]. For instance, a visible chest bruise may correspond to severe internal injuries like a ruptured diaphragm or punctured lung, which would otherwise go unnoticed.

The implementation of standard autopsy protocols is crucial for the thorough examination of thoracic trauma in road traffic accident victims. These protocols include systematic procedures to ensure a comprehensive evaluation of all potential injuries, which can confirm the cause of death and provide a complete injury assessment [18]. Standard protocols typically begin with an external examination, followed by a detailed internal examination of the thoracic cavity. This meticulous process helps identify and document injuries such as rib fractures, lung contusions, and cardiac lacerations, which are common in thoracic trauma cases. By adhering to these protocols, forensic pathologists can ensure consistent and accurate findings, which are essential for legal and medical purposes.

Special considerations are necessary when conducting thoracic examinations in autopsies, particularly in cases involving road traffic accidents. Given the high incidence of thoracic injuries in such accidents, which can be as high as 95%, pathologists must be vigilant in identifying various injury patterns [51]. These considerations include the need to carefully examine the thoracic cage for fractures and other structural damage, as well as inspecting the internal organs for signs of trauma. For instance, rib fractures were found in 43.7% of cases with fatal chest injuries, often accompanied by other injury combinations [52]. Additionally, the examination must account for both blunt and penetrating injuries, as these mechanisms can cause different types of damage to the thoracic cavity. This detailed approach ensures a thorough understanding of the trauma sustained and aids in uncovering any missed injuries that could have contributed to the overall cause of death.

Furthermore, especially in very violent accidents with extensive injury and with multiple passengers, there may be doubts about who was in the driver’s position and who was transported in the front and back of the vehicle. In these cases, the autopsy is crucial to determine the position of the occupants based on their injuries. Furthermore, there are often doubts as to whether or not the passengers were wearing seatbelts at the time of the accident. The autopsy is very useful in these cases, because it can identify typical signs of seatbelt such as infiltration of the subcutaneous or thoracic tissues and neck with an oblique distribution, which are not always present on external examination. In other cases, the autopsy is also essential to identify injuries related to the means of protection. In fact, it is known that the seatbelt and the explosion of the airbag can cause traumatic injuries to the thorax in high-energy accidents, such as a fracture of the sternum or rib fractures.

## 8. Diagnostic Techniques for Chest Injuries

The use of imaging and other diagnostic tools has significantly enhanced the accuracy and efficiency of postmortem diagnostics. Postmortem computed tomography (PMCT) and magnetic resonance imaging (MRI) have been particularly valuable in supplementing traditional autopsy methods. PMCT, for instance, is more sensitive than conventional autopsy in detecting skeletal injuries and abnormal gas accumulations, while traditional autopsy remains superior for identifying soft tissue and vascular injuries [47]. Diagnostic imaging tools are indispensable in the evaluation of chest trauma, contributing to the identification of injuries that might be missed during a physical examination.

Imaging methods such as X-rays, CT scans, and MRI play a crucial role in diagnosing chest injuries resulting from road traffic accidents. X-rays are often the first imaging technique used in emergency settings, due to their accessibility and rapid results. They are particularly effective in identifying rib fractures, foreign bodies, ballistic fragments, contusions, pneumothorax, hemothorax, and mediastinal abnormalities. However, CT scans provide a more detailed evaluation and are more precise than chest radiography in evaluating pulmonary contusions, thoracic aortic injury, and osseous trauma, especially in the cervicothoracic region. MRI, while less commonly used in acute trauma settings due to longer scanning times, offers superior soft tissue contrast and can be essential in evaluating complex injuries involving the chest wall, spine, and diaphragm [29]. While forensic autopsy is essential for determining the cause of death in road traffic accident victims, it could be difficult to identify certain thoracic injuries, especially subtle rib fractures, aortic dissections, or pneumothorax that could be better visualized through postmortem imaging techniques like PMCT. For example, studies have found that nearly 20% of rib fractures detected by PMCT were overlooked in standard autopsy [12]. This highlights the need for multimodal forensic analysis, integrating both radiological and autopsy assessments to ensure a complete understanding of trauma patterns in RTAs.

A thorough physical examination includes inspecting the chest for bruises, deformities, and open wounds; palpating for tenderness, crepitus, and subcutaneous emphysema; and auscultating for abnormal breath sounds such as decreased breath sounds, wheezing, or crackles. Additionally, obtaining a detailed clinical history is crucial, as it provides insight into the mechanism of injury and potential underlying conditions [28]. Transitioning from physical examination to imaging studies helps corroborate clinical findings and guides further management, ensuring a comprehensive approach to patient care.

The role of point-of-care ultrasound (POCUS) in emergency settings has become increasingly prominent in the rapid assessment of chest injuries. POCUS is a noninvasive, portable diagnostic tool that allows for the immediate evaluation of patients with hemodynamic instabilities. It is particularly valuable for identifying life-threatening conditions such as pneumothorax, hemothorax, and cardiac tamponade, which require prompt intervention. Bedside lung ultrasound, for instance, can lead to the rapid and accurate diagnosis of major pathologies in blunt chest trauma patients, thereby expediting the decision-making process for emergency interventions. The adoption of POCUS in emergency departments has the potential to revolutionize critical care by providing real-time diagnostic capabilities and enhancing the overall management of thoracic emergencies.

## 9. Preventive Measures for Chest Trauma

Proper seatbelt usage is paramount for minimizing the risk of chest injuries during vehicular accidents. Studies have shown that seatbelt use is associated with less severe injuries and lower in-hospital mortality rates, underscoring their critical role in passenger safety [41]. The seatbelt’s primary function is to restrain the occupant’s body, preventing it from striking the interior parts of the vehicle, such as the steering wheel or dashboard, which can lead to serious injuries. When used correctly, seatbelts distribute the force of impact across the stronger parts of the body, like the pelvis and ribcage, thereby reducing the likelihood of severe chest injuries.

Airbag deployment also plays a crucial role in reducing the severity of injuries during a crash. Airbags are designed to deploy upon impact, cushioning the blow and preventing the occupants from being thrown forward into hard surfaces within the car [53,54]. However, while airbags are effective in mitigating injuries, they can also cause chest injuries such as contusions or even fractures due to the force of their deployment [55]. For instance, there have been cases where individuals have sustained airbag-induced thoracic injuries, highlighting the potential harm that can occur despite the protective intent [56]. It is essential to understand that airbags work best in conjunction with seatbelts, as the combination provides a more comprehensive safety mechanism.

Modern vehicles are equipped with various safety features designed to protect occupants during collisions. These features include advanced seatbelt designs with pre-tensioners and load limiters, as well as sophisticated airbag systems that deploy strategically based on the severity of the crash. Additionally, vehicle manufacturers are continuously improving crashworthiness through better structural designs and materials that absorb impact energy more effectively. These innovations not only reduce the likelihood of chest injuries but also enhance overall occupant protection. For example, the use of a lap belt in combination with a deployed airbag has been shown to significantly reduce the incidence of flail chest in crashes, demonstrating the effectiveness of integrated safety systems [57].

## 10. The Need for Standardization in Forensic Protocols for Thoracic Trauma Analysis

Despite advancements in forensic pathology, the evaluation of thoracic trauma in road traffic fatalities remains inconsistent across different forensic institutions, leading to variability in injury documentation, interpretation, and accident reconstruction. Given the complexity of thoracic injuries and their role in determining cause of death, a standardized forensic protocol is essential for ensuring consistency, accuracy, and reproducibility in trauma analysis.

One major challenge in forensic investigations is the lack of uniformity in postmortem examination techniques. Some forensic centers rely solely on traditional autopsy, whereas others integrate postmortem computed tomography (PMCT) or MRI, resulting in differences in injury detection rates. Studies have demonstrated that PMCT enhances the identification of rib fractures and pneumothorax, which may go unnoticed in conventional autopsy. Therefore, forensic guidelines should mandate a combined approach using both autopsy and PMCT in suspected thoracic trauma cases to improve diagnostic accuracy.

Another crucial aspect of standardization involves injury classification and reporting. Different forensic pathologists may describe rib fractures and lung contusions using varying terminologies and grading scales, leading to potential discrepancies in forensic conclusions. Establishing a uniform injury classification system, similar to the Abbreviated Injury Scale (AIS) used in clinical trauma assessment, would facilitate cross-institutional comparisons and enhance forensic research.

To address these challenges, international forensic organizations should implement structured postmortem examination guidelines, incorporating the following:

- Mandatory PMCT screening for suspected thoracic trauma in RTA fatalities.
- Uniform terminology and injury classification protocols for rib fractures, lung injuries, and vascular trauma.
- Detailed forensic documentation of injury patterns to facilitate accident reconstruction and legal assessments.

By establishing these forensic standards, thoracic trauma investigations in RTAs will become more reliable, reproducible, and legally robust, ultimately improving forensic accuracy and ensuring that injury assessments are conducted with scientific precision and consistency across different jurisdictions.

## Data Availability

Not applicable to this article, as no datasets were generated.
